# Pet imaging of receptor occupancy

**DOI:** 10.1192/j.eurpsy.2021.39

**Published:** 2021-08-13

**Authors:** G. Knudsen

**Affiliations:** Neurobiology Research Unit 6931, Rigshospitalet and Univ. Copenhagen, Copenhagen O, Denmark

## Abstract

The discovery and development of drugs for treatment of brain disorders is an extremely challenging process requiring large resources, timelines, and associated costs. Positron Emission Tomography (PET) enables in vivo neuroimaging of various components of receptors, transporters, enzymatic activity and other types of proteins. PET also allows for studying the response to physiological or drug interventions in experimental medicine studies. Moreover, PET neuroimaging can assist to establish diagnoses in certain brain disorders and thereby improve patient selection and stratification for clinical trials. Over the past couple of decades, PET neuroimaging has thus become a central component of the evaluation of novel drugs for brain disorders, enabling decision-making in phase I studies, where early discharge of risk provides increased confidence to progress a candidate to a later phase testing at the right dose level or alternatively to kill a compound through failure to meet key criteria. The so called "3 pillars" of drug survival, namely; tissue exposure, target engagement, and pharmacologic activity, are particularly well suited for evaluation by PET imaging. Molecular neuroimaging has thus increasingly established itself as a unique tool that not only can demonstrate drug penetration and kinetics in the brain, but also identify pharmacodynamic effects, e.g., changes in glucose metabolism. It can also quantitate therapeutic action in vivo by determining, e.g., drug occupancy whereby the relevant dose ranges to be used in clinical efficacy trials can be determined.

**Disclosure:**

No significant relationships.

